# TET Enzymes as Epigenetic Integrators in Intestinal Immunity, Inflammation, and Disease

**DOI:** 10.3390/jpm16070375

**Published:** 2026-07-14

**Authors:** Dhirendra K. Singh, Yukihiro Yamaguchi, Lei Huang, Chieko Saito, Olivia G. Cassidy, Keita Nishiyama

**Affiliations:** 1 Department of Rheumatology and Inflammation Research, University of Gothenburg, 40530 Gothenburg, Sweden; dhirendra.singh.2@gu.se; 2 Department of Pediatrics, University of North Carolina at Chapel Hill, Chapel Hill, NC 27514, USA; 3 Faculty of Medical Sciences, Newcastle University, Newcastle upon Tyne NE2 4HH, UK; 4 Department of Biology, American University, Washington, DC 20016, USA; chiekosaito@gmail.com; 5 Laboratory of Animal Food Function, School of Agricultural Science, Tohoku University, Sendai 980-8572, Japan; keita.nishiyama.a6@tohoku.ac.jp

**Keywords:** TET1, TET2, TET3, Th17 Treg, DNA methylation, inflammatory bowel disease (IBD), colorectal cancer (CRC), Hirschsprung’s Disease (HSCR)

## Abstract

DNA methylation plays a fundamental role in maintaining intestinal homeostasis, immune tolerance, and inflammatory balance. Active DNA demethylation, mediated by the ten-eleven translocation family of dioxygenases (TET1, TET2, and TET3), has emerged as an important epigenetic mechanism linking environmental and metabolic cues to gene regulatory programs in the gut. In the intestinal epithelium, TET-dependent DNA hydroxymethylation contributes to intestinal stem cell maintenance, epithelial differentiation, regeneration, and barrier integrity. Perturbations in TET activity are associated with epithelial dysfunction, chronic inflammation, and increased susceptibility to colorectal tumorigenesis. Within the immune compartment, TET-mediated demethylation is required for the epigenetic stabilization of gut-associated immune cells. Altered TET function has been implicated in immune imbalance in inflammatory bowel disease, Hirschsprung’s disease, and colitis-associated colorectal cancer. Emerging evidence further indicates that intestinal microbiota-derived metabolites, including short-chain fatty acids and aryl hydrocarbon receptor ligands, modulate TET activity, positioning TET enzymes as epigenetic sensors of microbial and metabolic signals. In turn, TET-dependent programs shape immune responses to commensal microbes and pathogens, establishing a bidirectional microbiota–epigenetic axis that influences both intestinal and systemic immunity. In this review, we summarize and critically evaluate current evidence on the roles of TET enzymes in intestinal epithelial biology, immune cell regulation, and host–microbiota interactions in colorectal inflammation and disease.

## 1. Introduction

Epigenetic modifications, particularly DNA methylation, play a central role in regulating gene expression under both physiological and pathological conditions. DNA methylation typically involves the addition of a methyl group to the 5-position of cytosine residues within CpG dinucleotides and is generally associated with transcriptional repression. Aberrant DNA methylation, encompassing both hypermethylation and hypomethylation, is increasingly recognized as a key contributor to colorectal pathologies, influencing disease initiation, progression, and immune dysregulation [1,2]. Hypermethylation of tumor suppressor gene promoters represents a well-established hallmark of colorectal cancer (CRC), leading to gene silencing, uncontrolled cellular proliferation, resistance to apoptosis, and immune evasion [1]. Beyond oncogenesis, aberrant hypermethylation also affects genes involved in immune and inflammatory pathways, thereby exacerbating mucosal inflammation and tissue remodeling in inflammatory bowel disease (IBD), including ulcerative colitis (UC) and Crohn’s disease (CD) [3,4,5].

In contrast, DNA hypomethylation often reflects that active DNA demethylation has emerged as a complex epigenetic mechanism with important roles in inflammatory conditions of the colon. Loss of methylation at immune related and proinflammatory gene promoters can result in sustained activation of inflammatory pathways, enhanced immune cell infiltration, and prolonged tissue injury. Such changes have been documented in IBD, Hirschsprung’s disease (HSCR), and CRC [6,7,8,9]. In IBD, promoter hypomethylation of genes such as *TNF* correlates with increased expression in inflamed mucosa, reinforcing inflammatory signaling cascades [9]. Similarly, reduced methylation of *SOCS3*, a negative regulator of the JAK/STAT pathway, has been observed in CD and may amplify cytokine signaling and inflammatory responses [10]. Genome-wide analyses further reveal extensive methylation differences between inflamed and non-inflamed intestinal mucosa in IBD, underscoring their roles not only as biomarkers of disease activity but also as contributors to epithelial dysfunction, immune dysregulation, and impaired wound healing [9].

Active DNA demethylation is primarily mediated by the ten–eleven translocation (TET) family of enzymes TET1, TET2, and TET3 which catalyze the oxidation of 5-methylcytosine (5-mC) to 5-hydroxymethylcytosine (5-hmC), thereby facilitating base excision repair-dependent demethylation and gene reactivation. Dysregulation of TET enzymes in the intestinal epithelium is closely associated with barrier defects, microbial dysbiosis, chronic inflammation, and increased tumorigenic potential [6,11]. In addition, TET enzymes play a key role in translating environmental cues, particularly microbiota-derived metabolites such as short-chain fatty acids (e.g., butyrate)-into epigenetic reprogramming that governs gene expression and cellular homeostasis. Disruption of this metabolite-TET axis, either through altered microbial metabolism or impaired TET function, can promote inflammation, epithelial dysfunction, and tumorigenesis [12,13,14]. Beyond canonical DNA methylation, the identification of 5-hmC and its further oxidized derivatives has revealed additional layers of epigenetic regulation, highlighting the remarkable plasticity of the intestinal mucosa in response to environmental, microbial, and metabolic signals [15,16]. Collectively, these observations position TET mediated DNA demethylation as a pivotal mechanism in colorectal inflammation, HSCR, and CRC, and suggest its potential as a therapeutic target. While initially characterized as DNA demethylation enzymes, the broader literature shows that their functions extend into chromatin architecture, genome stability, immune regulation, and tumor suppression. A major unifying concept is that TET enzymes maintain genome integrity beyond CpG methylation control. Loss of TET function leads to heterochromatin instability, including heterochromatin-to-euchromatin switching, transcriptional readthrough, and derepression of repetitive elements [17,18,19].

In this review, we focus on the roles of TET enzymes in intestinal health and disease, with particular focus on their contributions to gene regulation, epithelial integrity, immune responses, and interactions with the intestinal microbiota.

## 2. Epigenetic Regulation of Intestinal Homeostasis and Inflammation by TET Enzymes

**Figure 1:**
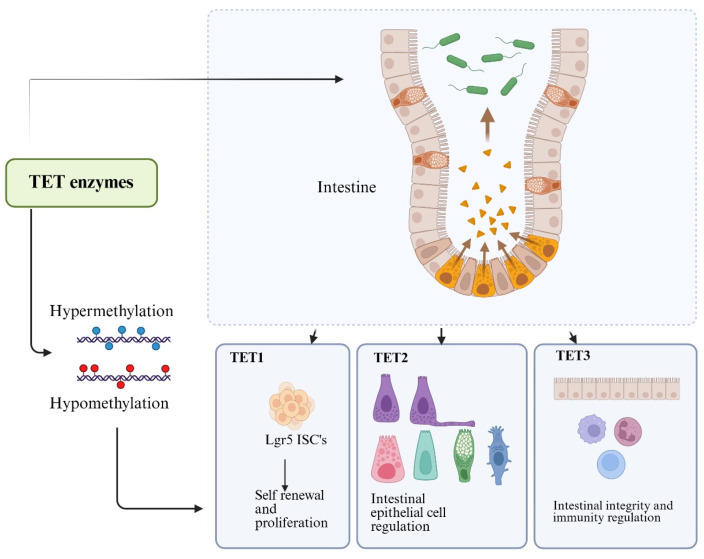
Functional roles of TET family enzymes in intestinal epigenetic regulation. TET enzymes control DNA methylation, i.e., hyper or hypomethylation to regulate gene activity in the intestine. In intestinal epithelium, TET1 supports Lgr5^+^ intestinal stem cell (ISC) self-renewal and proliferation, TET2 regulates epithelial lineage specification and homeostasis, and TET3 contributes to maintaining epithelial integrity and immune regulation.

DNA methylation is a central epigenetic modification regulating gene expression, development, and cellular identity. TETs (TET1, TET2, and TET3) mediate locus-specific oxidation of 5-mC to 5-hmC, enabling active DNA demethylation and epigenetic reprogramming essential for embryogenesis, lineage commitment, and environmental adaptation [20,21,22] (Figure 1). TET activity is regulated by multiple factors, including metabolic cofactors such as α-ketoglutarate, microbiota-derived short-chain fatty acids (SCFAs) (e.g., butyrate), recruitment by transcription factors, chromatin-associated proteins, and local chromatin accessibility. Together, these inputs allow context-dependent demethylation programs that are critical for epithelial differentiation, DNA repair, and immune cell polarization [20,21]. While TET1, TET2, and TET3 share common catalytic activity, they differ from one another in terms of genomic target recognition mechanisms, structural domains, and biological functions [23,24]. They exhibit isoform-specific expression patterns and functions within the intestine.

### 2.1. TET1

TET1 plays a central role in shaping transcriptional programs during development, the maintenance of tissue homeostasis, and disease processes [23,24,25]. In addition to its catalytic function, TET1 preferentially localizes to CpG-rich promoter regions. It serves as a crucial epigenetic regulator that stabilizes genome-wide methylation patterns while enabling context-dependent gene activation and repression [25]. TET1 accumulates primarily at promoter regions and transcription start sites in embryonic stem cells; this localization is essential for maintaining the naive pluripotent state and regulating gene expression during early development.

TET1 is preferentially expressed in Lgr5^+^ intestinal stem cells (ISCs) and adult stem cell populations, where it supports crypt homeostasis by depositing 5-hmC at Wnt target gene promoters, including *Lgr5* and *Axin2* [26,27,28]. Loss of TET1 impairs ISC proliferation, organoid formation, and epithelial regeneration [26]. During fetal development, TET1-mediated demethylation establishes region-specific DNA methylation landscapes in the gut epithelium that are maintained in human intestinal organoids [29]. Genome-wide 5-hmC profiling further indicates that TET1 regulates transcription factor occupancy at HNF4A and CDX2 binding sites, thereby coordinating colonocyte differentiation and barrier formation; dysregulation of these programs contributes to colorectal cancer development [30]. TET1 controls the differentiation and activation of innate lymphoid cells (ILCs) in the gut. Gut commensal bacteria produce specific metabolites that suppress TET1, creating a feedback loop that directs ILC differentiation and maintains local immune tolerance and intestinal homeostasis [31].

### 2.2. TET2

Unlike TET1 and TET3, TET2 lacks an intrinsic CXXC DNA-binding domain; consequently, its genomic targeting relies heavily on recruitment by interacting with proteins and transcription factors, rather than autonomous DNA binding, to enable locus-specific regulation of DNA methylation [23,32]. TET2 plays a prominent role in the regulation of hematopoiesis and is the most frequently mutated member of the TET family in hematologic malignancies, particularly myeloid neoplasms [33,34,35,36]. TET2 primarily binds gene bodies and enhancers and plays a key role in hematopoietic stem cell differentiation (blood cell formation). It is expressed continuously throughout various developmental stages, exhibiting high activity particularly in blood, immune, and neural tissues.

TET2 acts as a master regulator of host–microbe interactions by controlling bile acid metabolism in intestinal epithelial cells [37]. Deficiency in TET2 leads to an accumulation of specific bile acids such as hyocholic acid, which can cause dysbiosis by altering the abundance of beneficial bacteria like *Lactobacillus* and *Akkermansia* [37]. TET2 and TET3 play complementary and partially overlapping roles in intestinal epithelial cells (IECs). Combined loss of TET2 and TET3 disrupts epithelial differentiation, resulting in depletion of Paneth and tuft cells, expansion of enteroendocrine cells, aberrant methylation at cell fate-determining enhancers, microbial dysbiosis, intestinal inflammation, and increased mortality [38].

### 2.3. TET3

TET3 plays a central role in epigenetic reprogramming during development and tissue differentiation [23,24]. Within the TET family, TET3 is specifically responsible for the rapid and extensive demethylation that occurs during early embryonic development, exhibiting high activity during the formation of the zygote. Specifically, it is involved in the demethylation of the paternal genome and the initial epigenetic reset that establishes totipotency during development [39,40,41,42]. These studies have revealed that TET3 is a key factor driving the initial wave of epigenome reprogramming in mammalian development. TET3 plays a crucial role in early embryonic development; it is the variant that exhibits peak activity at the time of fertilization and is primarily responsible for the rapid, genome-wide demethylation of the paternal genome in the zygote.

TET3 acts as a critical regulator of the gut epithelial DNA methylome. By promoting the expression of genes involved in the Notch and Wnt signaling pathways, it directly maintains intestinal barrier function [6]. It is critical for the gut’s defense against luminal stressors, such as pathogenic infections or chemical stress (e.g., DSS-induced colitis). Therefore, deletion of TET3 increases susceptibility to gut damage and inflammation [6,38,43]. TET3 is often working alongside TET2 and alters methylation at enhancer regions, controlling transcription factors that dictate cell fate in the small intestine. Its absence leads to a severe loss of mature Paneth cells which secrete antimicrobial peptides and an imbalance in enteroendocrine cell types [24,38].

Environmental exposure to Perfluorooctanoic acid (PFOA) can disrupt the epigenetic machinery that maintains intestinal gene regulation. PFOA exposure is associated with decreased expression of key DNA methylation regulators, including TET enzymes, particularly TET3, in intestinal tissues. This reduction in TET3 may impair normal DNA demethylation dynamics, contributing to widespread epigenetic imbalance. Concurrently, PFOA alters tight junction gene expression in a dose- and tissue-dependent manner, indicating compromised epithelial barrier regulation. Together, these changes link environmental toxicity to disrupted TET3-dependent epigenetic control of intestinal homeostasis [44]. These findings highlight the sensitivity of TET-dependent epigenetic networks to external insults and underscore their relevance in intestinal disease pathogenesis.

### 2.4. Intestinal Immune Cells

Genome-wide analyses have demonstrated that 5-hmC is enriched at active genes and enhancer regions during T-cell development, establishing TET enzymes as key regulators of lineage-specific transcriptional programs [45]. In addition to DNA demethylation, TET proteins regulate chromatin accessibility, enhancer activation, genomic stability, and transcriptional fidelity across diverse immune cell populations [46,47,48].

#### 2.4.1. Roles of TETs in Th17 and Regulatory T Cells

TET enzymes are central regulators of CD4^+^ T cell fate, orchestrating the balance between inflammatory Th17 cells and immunosuppressive regulatory T cells (Tregs) through DNA demethylation. Genome-wide analyses have shown that TET2-mediated generation of 5-hmC is enriched at lineage-specific cytokine loci, including *IL-17A/F*, where it promotes transcriptional activation via enhancer-associated DNA demethylation, thereby supporting Th17 effector function [49]. At the same time, TET activity is tightly regulated by cellular metabolism; the immunometabolite 2-hydroxyglutarate (2-HG), produced through the Got1-α-ketoglutarate (α-KG) pathway, inhibits TET-dependent DNA demethylation, leading to sustained methylation of the *FOXP3* locus and stabilization of the Th17 phenotype [50]. Conversely, reduction of 2-HG restores TET activity, promotes *FOXP3* demethylation, and facilitates the reprogramming of Th17 cells toward a regulatory phenotype, highlighting the integration of metabolic and epigenetic signals in controlling T cell plasticity.

In parallel, TET enzymes are indispensable for maintaining Treg lineage stability and immune tolerance by enforcing epigenetic accessibility at the *FOXP3* locus. DNA methylation at *FOXP3*, particularly within the Treg-specific demethylated region (TSDR), critically determines Treg stability and suppressive function [51,52,53,54]. TET1 and TET2 are recruited to the *FOXP3* locus downstream of TGF-β/Smad3 and IL-2/STAT5 signaling, where they mediate active DNA demethylation and sustain stable Foxp3 expression; disruption of this process results in *FOXP3* hypermethylation, impaired Treg function, and autoimmunity [55]. Similarly, combined deficiency of TET2 and TET3 leads to hypermethylation of the TSDR, loss of Foxp3 expression, and conversion of Tregs into pro-inflammatory, Th17-like effector cells, culminating in systemic inflammation [56]. Notably, dysregulated TET activity can also perturb immune homeostasis, as excessive TET2 activity in certain inflammatory contexts may disrupt the Th17/Treg balance, whereas its modulation can restore Treg populations [57]. Therapeutically, strategies targeting upstream regulators such as RORC2 suppress Th17 programs while preserving *FOXP3* demethylation and Treg stability [58], and emerging approaches such as CRISPR–TET1-mediated epigenetic editing of the TSDR further underscore the potential of TET-based interventions to reinforce immune tolerance [59,60]. Collectively, these findings establish TET enzymes as key integrators of metabolic and epigenetic cues that govern Th17/Treg plasticity and highlight their importance as therapeutic targets in inflammatory diseases such as IBD.

#### 2.4.2. T Cell Subsets and iNKT Cells

In invariant natural killer T (iNKT) cells, the combined loss of Tet2 and Tet3 impairs lineage commitment and promotes aberrant differentiation into NKT17 cells, while also causing reduced expression of T-bet and Th-POK and triggering uncontrolled proliferation [61]. Mechanistically, TET-mediated demethylation influences PLZF expression and iNKT cell maturation by promoting GATA3 binding to the Zbtb7b (Th-POK) locus and regulating Drosha-mediated microRNA biosynthesis [62,63]. Furthermore, TET deficiency leads to genomic instability, aneuploidy, elevated Myc expression, and a loss of T-cell receptor repertoire diversity, highlighting the critical role of TET enzymes in maintaining clonal homeostasis and genomic integrity [64,65,66]. Recent studies have also revealed that TET enzymes serve as comprehensive guardians of chromatin structure and genomic stability; the loss of TET activity disrupts heterochromatin organization, promotes the conversion of heterochromatin to euchromatin, activates repetitive sequences and transposons, and increases transcriptional read-through [17,18,19]. These abnormalities lead to the accumulation of R-loops and G-quadruplex structures, DNA damage, and aberrant chromosome segregation, culminating in the rapid onset of myeloid malignancies [67,68,69]. TET dysfunction can arise from genetic mutations or metabolic inhibition by oncometabolites such as 2-hydroxyglutarate; additionally, OGT-dependent regulation further modulates TET activity and heterochromatin stability [19,69,70].

#### 2.4.3. Macrophages

TET1 has been shown to promote M1-like (pro-inflammatory) macrophage polarization in an in vitro system by enhancing NF-κB signaling and increasing TNFα production in experimental macrophage models [71,72]. Knockdown or inhibition of TET1 attenuates these proinflammatory responses, indicating a non-redundant role for TET1 in macrophage activation, with limited compensatory activity from other TET isoforms in these settings.

Collectively, TET enzymes integrate metabolic, microbial, and environmental signals to orchestrate intestinal stem cell maintenance, epithelial differentiation, immune modulation, and host–microbe interactions (Table 1 and Table 2). Disruption of TET function contributes to epithelial dysfunction, chronic inflammation, and increased cancer risk, positioning TET regulated pathways as promising targets for therapeutic intervention in intestinal diseases.

## 3. TET Enzymes in Intestinal Disorders

TET enzymes, particularly TET1 and TET2, function as key epigenetic regulators that contribute to gut homeostasis and shape immune responses under inflammatory conditions. Dysregulation of TET-dependent DNA demethylation has been increasingly implicated in immune imbalance and intestinal pathology.

### 3.1. TET2 in Inflammatory Bowel Disease (IBD)

IBD is a chronic relapsing inflammatory disorder of the gastrointestinal tract characterized by dysregulated immune responses, epithelial barrier dysfunction, and altered host–microbiota interactions. Epigenetic mechanisms, including DNA methylation dynamics, are increasingly recognized as key contributors to disease susceptibility and progression.

Interestingly, TET2 expression is often increased during experimental inflammatory responses, whereas intestinal tissues from patients with active IBD frequently exhibit reduced TET2 levels, suggesting that dysregulated TET2 activity contributes to disease pathogenesis [7]. Consistent with this notion, TET2 deficiency exacerbates colitis severity and impairs epithelial repair mechanisms. Moreover, TET2 expression inversely correlates with connexin 43 (Cx43), indicating potential feedback regulation between epigenetic control and gap junction–mediated signaling pathways [7]. Pharmacological targeting of TET2-dependent pathways further supports its functional relevance, as compounds such as the TET inhibitor IOX1 suppress Th17-driven inflammatory responses, underscoring the therapeutic potential of modulating TET2 activity in IBD [82].

### 3.2. Roles of α-KG in IBD

TET enzymes require α-KG [83], a product of the tricarboxylic acid (TCA) cycle and as co-factors for their catalytic activity. Citrate converts to isocitrate, which then changes to α-KG through isocitrate dehydrogenase (IDH). This process generates reduced NAD^+^, which the electron transport chain uses to produce ATP and energy. TET activity relies on both IDH function and α-KG levels. Research has shown that mutations in IDH lead to the production of 2-hydroxyglutarate instead of α-KG, resulting in decreased TET activity. This effect is observed in some cancers [84]. The reliance of TET on metabolites from the citric acid cycle illustrates one of many ways cellular metabolism can impact epigenetic processes. The balance of TCA cycle metabolites acts as a metabolic regulator for epigenetic enzyme activity. This has important implications for inflammatory changes in IBD. In addition to regulating epigenetics, α-KG has direct protective roles in maintaining intestinal health. It helps support the integrity of the epithelium, lower oxidative stress, and adjust inflammatory signaling. It promotes the recovery of epithelial cells via Wnt/β-catenin signaling while alleviating endoplasmic reticulum stress. This helps restore barrier function in models of colitis [85]. Furthermore, α-KG improves how the epithelium processes energy by shifting metabolism toward oxidative phosphorylation rather than glycolysis. This shift is linked to lower inflammation and better mucosal integrity [86]. Its roles also include being an anti-inflammatory and antioxidant metabolite that helps cells respond to stress [87]. Moreover, α-KG and its derivative, ornithine α-ketoglutarate (OKG), affect immune and microbial environments in the gut. They promote anti-inflammatory macrophage activity, reduce the production of pro-inflammatory cytokines, and boost antioxidant defenses, all of which aid in maintaining immune balance [86,88]. They also change the gut microbiome by increasing beneficial bacteria like *Lactobacillus* and *Alistipes* while decreasing harmful ones, improving intestinal barrier function and lowering inflammation [88,89]. These findings support a coherent model where problems in mitochondrial TCA cycle function and lower levels of α-ketoglutarate contribute to both epigenetic issues and compromised intestinal health in IBD. α-KG serves as both a metabolic building block and an epigenetic cofactor, connecting mitochondrial energy metabolism to DNA demethylation through TET enzymes. At the same time, it regulates epithelial repair, immune function, and the composition of microbiota. Therefore, targeting the TCA cycle-α-KG-TET pathway may offer a promising treatment approach for restoring metabolic and epigenetic balance in inflammatory bowel diseases [85,86,87,88,90,91].

### 3.3. Colorectal Cancer (CRC)

Accumulating evidence indicates that TET1 and TET2 function as important epigenetic regulators that restrain colorectal tumorigenesis by maintaining appropriate DNA methylation landscapes at tumor suppressor and DNA repair genes. In colorectal cancer (CRC), disruption of TET1 activity is associated with altered 5-hmC distribution and dysregulated gene expression programs that facilitate tumor progression [30]. Chronic inflammatory conditions, including IBD, as well as microbiota-driven alterations in TET-dependent DNA methylation, may further contribute to oncogenic transformation in the colon [92].

TET2 plays a particularly prominent role in preserving promoter hypomethylation of key tumor suppressor genes, including *MLH1*. Loss of AMP-activated protein kinase (AMPK) activity reduces intracellular α-KG availability and impairs TET2 function, leading to hypermethylation of tumor suppressor genes, defective DNA mismatch repair, and accelerated CRC progression [11,76]. Collectively, these findings support a tumor-suppressive role for TET2 and suggest that restoring TET activity or its metabolic cofactors may represent a potential therapeutic strategy, particularly in colitis-associated CRC [7,92].

### 3.4. Emerging Roles of SATB1 in Intestinal Immunity and Epigenetic Control

Alongside TET enzymes, SATB1 (Special AT-rich Sequence Binding Protein 1) has been identified as a “master chromatin organizer” that regulates higher-order genome structure and governs transcriptional programs in immune cells. SATB1 influences tissue adaptation, inflammatory signaling, and T-cell differentiation in a highly context-dependent manner [93,94,95,96,97,98]. In the intestine, SATB1 contributes to the establishment of compartment-specific transcriptional programs in CD4^+^ T cells and Treg cells residing in the epithelium and lamina propria, thereby regulating local immune responses during homeostasis and in IBD [97]. Furthermore, regulatory pathways centered on SATB1 modulate inflammatory responses through long non-coding RNA (lncRNA) networks. For instance, the ANRIL/miR-191-5p/SATB1 axis suppresses the production of IL-6 and TNF-α, exerting a protective effect against experimental colitis [98]. Similar anti-inflammatory functions mediated by SATB1-associated lncRNAs, which regulate oxidative stress and cytokine production, have also been observed in other tissues [99]. Recent research has further revealed that SATB1 plays a crucial role in Th17 cell differentiation and pathogenic immune responses. Functioning as a pioneer chromatin factor, SATB1 remodels chromatin accessibility at *Il2* locus to suppress IL-2 signaling while simultaneously promoting lineage commitment to Th17 cells [100]. Given that *Satb1* deficiency inhibits Th17 cell differentiation and confers protection against Th17-mediated autoimmune diseases, SATB1 is recognized as a key regulator of the inflammatory T-cell program. SATB1 is also involved in innate immunity; following macrophage activation, it acts in concert with HDAC1 to regulate the expression of IFN-β, IRF7, STAT1, TNFα, and other inflammatory mediators [101]. Thus, SATB1 functions as a chromatin-based regulator that controls both adaptive and innate immune responses. A particularly significant mechanistic advancement is the recent discovery of a direct interaction between SATB1 and DNA methylation pathways. In a study utilizing a model of mature T-cell-specific deficiency, Seo et al. demonstrated that the loss of *Satb1* leads to the spontaneous emergence of atypical FoxP3^+^CD25^−^ cells from naive conventional CD4^+^ T cells. This aberrant Foxp3 expression occurs independently of TGF-β signaling and relies on TET2- and TET3-mediated DNA demethylation. While Tet2/Tet3 deficiency suppresses the derepression (release from transcriptional silencing) of Foxp3, the loss of DNMT1 further enhances it; this indicates that SATB1 maintains the epigenetic silencing of Foxp3 by regulating DNA methylation [102]. Importantly, the FoxP3^+^ cells generated in this manner are unstable and fail to acquire full Treg functionality. This suggests that SATB1 acts as an epigenetic “gatekeeper,” preserving the identity of conventional CD4^+^ T cells by preventing the inappropriate activation of the Treg program.

## 4. Microbiota–TET Axis

**Figure 2:**
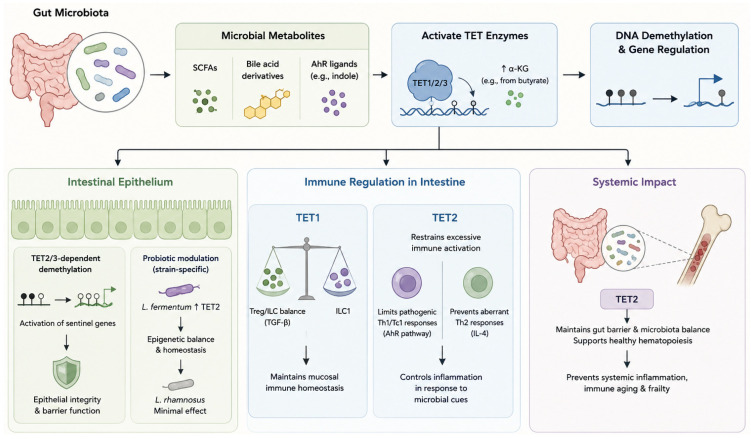
Microbiota-dependent regulation of TET enzymes in intestinal homeostasis. Microbial metabolites, including SCFAs, bile acid derivatives, and AhR ligands, regulate TET1/2/3 activity and DNA demethylation. TET-dependent epigenetic remodeling promotes epithelial barrier integrity, probiotic-mediated homeostasis, and balanced immune responses. TET1 maintains innate lymphoid cell homeostasis, whereas TET2 restrains excessive inflammation and supports gut–bone marrow communication. Dysregulation of the microbiota–TET axis contributes to intestinal inflammation, immune dysfunction, aging-associated inflammation, and tumorigenesis.

The intestinal microbiota exerts a major influence on host epigenetic regulation by shaping the availability of metabolites and cofactors that directly modulate DNA methylation and demethylation pathways. Microbial derived metabolites including SCFAs, bile acid derivatives, and aryl hydrocarbon receptor ligands-alter cellular metabolic states and chromatin-associated enzyme activity, thereby influencing TET dependent DNA demethylation programs in intestinal epithelial and immune cells (Figure 2 and Table 2). Through these mechanisms, microbiota contributes to dynamic regulation of gene expression networks that govern epithelial integrity, immune tolerance, and inflammatory responses. Dysregulation of microbiota-TET interactions has been increasingly implicated in intestinal inflammation, immune imbalance, and colorectal tumorigenesis.

### 4.1. SCFAs as Metabolic Modulators of TET Activity

SCFAs, particularly butyrate, influence host epigenetic regulation by modulating cellular metabolism and the activity of TET dioxygenases. Butyrate increases intracellular levels of α-ketoglutarate, an essential cofactor for TET enzymes, thereby promoting active DNA demethylation. In experimental models, butyrate-induced activation of TET-dependent pathways enhances demethylation of promoters of mismatch repair genes, including *MLH1*. This is associated with improved DNA repair capacity and reduced susceptibility to colorectal tumorigenesis, and TET supports a model in which butyrate may enhance TET-dependent demethylation [73]. These findings illustrate how microbiota-derived metabolites can shape host epigenetic landscapes through metabolic control of TET enzyme activity.

### 4.2. Microbiota-Dependent TET2/3 Regulation in Intestinal Epithelial Cells

Microbial colonization induces TET2- and TET3-dependent DNA demethylation at enhancer and promoter regions in intestinal epithelial cells, leading to activation of sentinel gene programs that preserve epithelial integrity and restrain inflammatory responses [74]. In contrast, germ-free mice display distinct DNA methylation landscapes characterized by reduced demethylation at these regulatory elements, highlighting the requirement of microbiota-derived signals for TET2/3-mediated epigenetic programming in the intestinal epithelium [74].

### 4.3. Probiotic Mediated Modulation of TET Enzyme and Intestinal Epigenetics

Emerging evidence suggests that probiotic strains can differentially influence TET enzyme activity and broader host epigenetic regulation in a strain-specific manner. In intestinal epithelial cell models, *Limosilactobacillus fermentum* upregulates TET2, DNA methyltransferases (DNMTs), and histone-modifying enzymes, and can partially reverse *Escherichia coli*-induced suppression of these epigenetic regulators in Caco-2 which is a human epithelial cell line derived from a colorectal adenocarcinoma patient [81]. In contrast, *Lacticaseibacillus rhamnosus* exhibits minimal effects on host epigenetic gene expression in the same experimental setting [81]. These observations indicate that specific probiotic strains may modulate TET-dependent epigenetic programs in intestinal epithelial cells, highlighting a potential mechanism through which defined microbial interventions could influence epithelial homeostasis and immune regulation.

### 4.4. Microbiota–TET Regulation of Innate Immunity in Intestinal Homeostasis

Microbiota-derived signals regulate innate and adaptive immune balance through TET-dependent epigenetic mechanisms. In the intestine, TET1 restricts type 1 innate lymphoid cell (ILC1) differentiation by promoting TGF-β signaling, thereby maintaining ILC subset balance. Microbiota-driven reductions in colonic cholic acid decrease TET1 expression, leading to ILC1 expansion and disruption of mucosal immune homeostasis [31]. In parallel, TET2 restrains excessive immune activation in response to microbial cues. In the liver, *Tet2* deficiency promotes aryl hydrocarbon receptor (AhR)-dependent differentiation of IFNγ producing Tc1 cells, driven by dysbiosis-associated expansion of *Lactobacillus reuteri* and increased production of the AhR ligand indole-3-aldehyde, resulting in autoimmune hepatitis-like pathology [77]. In the intestine, loss of TET2 permits autonomous IL-4 production by naïve CD4^+^ T cells, promoting chronic Th2-driven inflammation in response to commensal protists such as *Tritrichomonas* [78].

Beyond local immune regulation, microbiota-TET2 interactions influence systemic immunity and hematopoiesis. In mouse models, *Tet2* mutations impair intestinal barrier integrity, induce microbial dysbiosis, and reshape the bone marrow niche, promoting preleukemic myeloproliferation and revealing a microbiota-dependent gut-bone marrow axis [79]. Consistent with these findings, human multi-omics and Mendelian randomization studies identify TET2 as a key node linking intestinal microbiota composition to systemic inflammation, immune aging, and frailty, highlighting its broader role in maintaining immune homeostasis across tissues [80].

## 5. Personalized Medicine in IBD and CRC

In IBD, analyzing DNA methylation profiles in blood or mucosal tissue enables the identification of acute inflammation. As active inflammation resolves with treatment, these methylation patterns tend to revert to baseline states; consequently, they have been established as markers for monitoring therapeutic efficacy and mucosal healing [103,104,105,106]. Patients with long-standing IBD face an increased risk of developing colitis-associated cancer (CAC). Research is actively underway to identify specific epigenetic changes, including alterations in genes involved in TET/TDG-mediated active demethylation, that characterize patients progressing toward dysplasia and requiring closer endoscopic surveillance [43,107,108,109].

In CRC, alterations in DNA methylation profiles, such as the hypermethylation of specific CpG islands and a global reduction in 5-hmC, are widely recognized as hallmark features of the disease. Leveraging these markers in liquid biopsies (e.g., analyzing cfDNA in blood or stool) has led to the development of highly specific diagnostic methods for early-stage CRC [110,111,112,113]. Reduced TET expression and the consequent decline in downstream 5-hmC levels correlate with disrupted gene regulation, tumor progression, and shortened overall survival [30,114]. Furthermore, emerging evidence indicates that specific DNA methylation profiles, including those associated with tumor mutational burden, are useful for predicting patient responses to immune checkpoint inhibitors (ICIs) and certain chemotherapy regimens [115,116].

TET expression patterns, 5-hmC signatures, and DNA methylation profiles hold immense potential as clinical biomarkers for CRC and IBD, and their transition toward clinical application is rapidly advancing [1,43]. These epigenetic markers reflect the progression of cells from homeostasis to inflammation and, ultimately, tumorigenesis, demonstrating significant clinical utility [1]. However, many of these markers remain in the research stage and require further validation through large-scale prospective cohort studies.

## 6. Limitations and Future Directions

While accumulating evidence indicates that DNA methylation plays a pivotal role in the pathogenesis of IBD, several challenges remain that hinder the elucidation of underlying mechanisms and clinical application. Much current research relies on cross-sectional analyses of bulk tissue or peripheral blood, making it difficult to distinguish whether epigenetic changes are “drivers” (causes) or “consequences” of inflammation. Discrepancies across studies regarding specific methylation sites or the direction of regulation likely reflect differences in patient populations, disease stages, tissue sample types, and analytical methods. Furthermore, despite the highly cell-type-specific nature of DNA methylation, many studies lack the resolution required to analyze epigenetic heterogeneity in detail, particularly within intestinal epithelial cells and immune cell subsets such as tissue-resident macrophages and infiltrating inflammatory macrophages. Common experimental models, such as DSS-induced colitis and LPS-stimulated cell systems, are useful for mechanistic studies but fail to fully recapitulate the characteristic “chronic relapsing-remitting” course of human IBD. Additionally, currently available epigenetic drugs such as 5-azacytidine and decitabine induce genome-wide demethylation; this raises concerns regarding off-target effects, disruption of immune homeostasis, and uncertain long-term safety, thereby limiting their therapeutic utility [117,118,119].

These issues also point to important directions for future research. Integrating single-cell sequencing, single-cell methylomics, spatial transcriptomics, and multi-omics analysis is expected to enhance the resolution of cell-type-specific methylation programs and elucidate the dynamic interactions between epithelial and immune cells during intestinal inflammation. Establishing the temporal sequence and causal relationships linking DNA methylation changes to immune activation and disease progression requires longitudinal cohort studies and animal models that track changes over time. From a translational perspective, future therapeutic strategies may evolve from broad immunosuppression toward precision epigenetic and immune modulation. CRISPR/dCas9-based epigenome editing technologies, combining functional domains of DNMTs and TETs, enable targeted methylation or demethylation of specific regions within inflammation-related genes; furthermore, by selectively targeting individual DNMT or TET isoforms, these technologies could achieve greater specificity than conventional, broad-spectrum methylation inhibitors. Moreover, integrating DNA methylation signatures with immune cell states, microbiome profiles, and clinical phenotypes is expected to facilitate patient stratification, the prediction of treatment responses, and the identification of individuals at high risk of relapse, thereby contributing to the advancement of precision medicine for IBD.

## Figures and Tables

**Table 1:** TET Enzyme Functions in Gut Immunity and Disease.

TET Enzyme	Function	Gut Context	Key Outcomes	References
TET1	Suppresses ILC1, enhances TGF-β	ILC homeostasis	Prevents ILC1-driven inflammation	[31]
TET2	Modulates MyD88, Cx43, inflammasome	IBD, colitis, cancer	Immune regulation, DNA repair	[7,38]
TET3	Maintains 5-hmC, Paneth cell function	DSS colitis, infection	Barrier regeneration, Wnt activation	[6]
All	Modulated by microbiota and α-KG	Host–microbe epigenetic interface	Epigenetic programming	[73,74]

**Table 2:** Microbiota–TET Enzyme Interactions and Their Functional Impacts.

TET Enzyme	Biological Process/Target	Microbiota/Metabolite Influence	Functional Outcome	References
TET1	ILC1 differentiation	Microbiota reduce colonic cholic acid → downregulates TET1	Increased ILC1 differentiation; potential for inflammatory imbalance	[31]
TET1	NF-κB activation in macrophages	N/A	Promotes M1 polarization and TNFα production	[71,72]
TET1	Epigenetic regulation in gut injury	Glutamine and probiotics modulate TET1 via iNOS methylation	Reduced oxidative stress and inflammation	[75]
TET2	DNA mismatch repair gene (MLH1) expression	Butyrate ↑ α-KG → enhances TET2 activity; AMPK deficiency ↓ α-KG and TET2	Promotes DNA repair and suppresses tumorigenesis; deficiency leads to hypermethylation and CRC progression	[11,73,76]
TET2	Epigenetic reprogramming in epithelial cells	Microbiota colonization induces TET2/3-mediated demethylation at sentinel gene loci	Maintains epithelial homeostasis and immune regulation	[74]
TET2	CD8^+^ Tc1-mediated autoimmune hepatitis	Dysbiosis ↑ AhR-ligand-producing pathobionts (e.g., *L. reuteri*) in TET2-deficient hosts	Induces liver autoimmunity	[77]
TET2	Th2 inflammation via IL-4 production	Commensal *Tritrichomonas* in TET2-deficient T cells	Promotes chronic Th2 responses and barrier dysfunction	[78]
TET2	Hematopoiesis and myeloproliferation	Tet2 mutation disrupts gut barrier, modifies microbiota, and affects systemic metabolism	Preleukemic myeloproliferation	[79]
TET2	Frailty and aging	Microbiota composition influences TET2 expression and immune aging	Links dysbiosis to frailty via immune dysregulation	[80]
TET2	Probiotic-mediated gene modulation in IECs	*L. fermentum* ↑ TET2 expression; *E. coli* ↑ expression	Supports probiotic-based epigenetic therapy	[81]
TET3	Epithelial regeneration, immune response	DSS and pathogen exposure modulate TET3 activity and 5-hmC levels	TET3 loss leads to severe colitis, impaired Paneth cell function, and compromised autophagy	[6]
TET3	DNA demethylation at regulatory elements	Microbiota colonization required for TET3-mediated epigenetic programming	Regulates inflammation and colorectal cancer susceptibility	[74]
TET2/TET3	Genome-wide methylation landscape	Germ-free vs. conventional mice reveal microbiota-TET2/3 dependency	Critical for homeostatic gene expression under both normal and inflammatory conditions	[74]

“↑” means “increase” and “↓” means “decrease”.

## Data Availability

No new data were created or analyzed in this study. Data sharing is not applicable to this article.

## Abbreviations

5-hmC	5-hydroxymethylcytosine
5-mC	5-methylcytosine
AhR	aryl hydrocarbon receptor
α-KG	α-ketoglutarate
AMPK	adenosine monophosphate-activated protein kinase
ANRIL	antisense non-coding RNA in the INK4 locus
ATP	adenosine triphosphate
Axin2	axin-like protein (Axil), axis inhibition protein 2
CAC	colitis-associated cancer
Caco-2	cancer coli-2
CD	Crohn’s disease
CD4	cluster of differentiation 4
CD8	cluster of differentiation 8
CD25	cluster of differentiation 25
cDNA	complementary deoxyribonucleic acid
CDX2	Caudal type homeobox 2
CNS2	conserved non-coding sequence 2
CpG	5′-cytosine nucleotide-phosphate-guanine nucleotide-3′
CRC	colorectal cancer
CRISPR	clustered regularly interspaced short palindromic repeats
Cx43	connexin 43
dCas9	nuclease-dead CRISPR-associated protein 9
DNA	deoxyribonucleic acid
DNMT1	DNA methyltransferase 1
DNMT3b	DNA methyltransferase 3b
DNMTs	DNA methyltransfes
DSS	dextran sulfate sodium
*E. coli*	*Escherichia coli*
Foxp3	Forkhead box protein P3
GATA3	GATA binding protein 3
GDNF	glial cell line-derived neurotrophic factor
GFRA4	GDNF family receptor alpha 4
HDAC1	histone deacetylase 1
H_2_S	hydrogen sulfide
HNF4A	hepatocyte nuclear factor 4 alpha
HSCR	Hirschsprung’s disease
IBD	inflammatory bowel diseases
ICIs	immune checkpoint inhibitors
IDH	isocitrate dehydrogenase
IEC	intestinal epithelial cell
IFN β	interferon beta
IFNγ	interferon gamma
IL-17	interleukin-17
IL-2	interleukin-2
IL-4	interleukin-4
ILC	innate lymphoid cells
ILC1	type 1 innate lymphoid cell
ILC3	type 1 innate lymphoid cell
iNKT	invariant natural killer T
iNOS	inducible nitric oxide synthase
IOX1	5-carboxy-8-hydroxyquinoline
IRF7	interferon regulatory factor 7
ISC	intestinal stem cell
JAK	Janus kinase
Lgr5	Leucine-rich repeat-containing G-protein coupled receptor 5
lncRNA	long non-coding RNA
miR-191-5p	homo sapiens microRNA 191-5p
MLH1	mutL homolog 1
Myc	cellular myelocytomatosis oncogene
NAD^+^	nicotinamide adenine dinucleotide
NCC	neural crest cell
NFYB	nuclear transcription factor Y subunit-β
NF-κB	nuclear factor κ-light-chain-enhancer of activated B cells
NKT17 cells	NKT17: natural killer T-17 cells
OGT:	O-GlcNAc transferase
OKG:	ornithine α-ketoglutarate
PFOA	perfluorooctanoic acid
PLZF	Promyelocytic Leukemia Zinc Finger:
RET	rearranged during transfection
RNA	ribonucleic acid
SATB1	Special AT-rich Sequence Binding Protein 1
SCFAs	short-chain fatty acids
Smad3	Mothers against decapentaplegic homolog 3
SOCS3	Suppressor of Cytokine Signaling 3
STAT	signal transducer and activator of transcription
Stat1	signal transducer and activator of transcription 1
Stat5	signal transducer and activator of transcription 5
T-bet	T-box expressed in T cells/T-box transcription factor 21
Tc1	type I cytotoxic T cells
TCA cycle	tricarboxylic acid cycle
TDG	thymine DNA glycosylase
TET1	ten-eleven translocation enzyme 1
TET2	ten-eleven translocation enzyme 2
TET3	ten-eleven translocation enzyme 3
TGF-β	transforming Growth Factor-β
Th1	type 1 T helper cells
Th17	T helper 17 cells
Th2	type 2 T helper cells
ThPOK	T-helper inducing POZ/Krüppel-like factor
TNF	Tumor Necrosis Factor
TNF	Tumor Necrosis Factor alpha
Treg	regulatory T cell
TSDR	Treg-specific demethylated region
UC	ulcerative colitis
VKH	Vogt–Koyanagi–Harada disease
Zbtb7b	zinc finger and BTB domain containing 7B:
